# Low-dose statins combined with repetitive transcranial magnetic stimulation reduce post-stroke depression

**DOI:** 10.3389/fneur.2025.1649263

**Published:** 2025-11-26

**Authors:** Chaohua Cui, Chuhua Cai, Qiulian Yin, Haoye Guan, Tonghua Long

**Affiliations:** 1 Life Science and Clinical Medicine Research Center, Affiliated Hospital of Youjiang Medical University for Nationalities, Baise, Guangxi, China; 2 Drug Clinical Trial Management Office, The Second Affiliated Hospital Of Guangxi University of Science and Technology, Liuzhou, Guangxi, China

**Keywords:** post-stroke depression, statins, repetitive transcranial magnetic stimulation, incidence rate and prognosis, machine learning

## Abstract

**Background:**

The effectiveness of statins in preventing post-stroke depression (PSD) remains controversial. Lower testosterone levels caused by statins could potentially increase the risk of PSD. Low-dose statins could offer better benefits due to their lesser impact on testosterone levels. Repetitive transcranial magnetic stimulation (rTMS) has been shown to reduce PSD in the acute stage. The combination of low-dose statins and rTMS may provide a promising therapeutic option for patients with PSD.

**Methods:**

Data were collected from ischemic stroke patients. Unsupervised machine learning methods were employed to explore the risk factor of PSD. Our prospective cohort study collected data from ischemic stroke patients over 3 years. The patients’ conditions determined the prescription of statins. The outcomes measured included the incidence of PSD, favorable functional outcome (FFO) at 6- and 12-months post-onset.

**Results:**

A total of 545 patients were included in the study. The mean age was 60.99 ± 12.768 years, and 31.9% of the participants were female. The combination of low-dose statins and rTMS subgroup had the lowest incidence of PSD at both 6 and 12 months (*p* < 0.001). In multivariable logistic regression, the combination of low-dose statins and rTMS was inversely related to PSD at 6 months (OR = 0.370, *p* = 0.003) and 12 months (OR = 0.386, *p* = 0.004).

**Conclusion:**

The combination of statins and the rTMS group related the lower incidence of PSD in stroke patients. Combining low-dose statins and rTMS could reduce PSD and further improve the prognosis of ischemic stroke patients.

## Introduction

Post-stroke depression (PSD) is a common complication among ischemic stroke patients. These patients have worse functional outcomes and higher mortality rates than those without PSD (1). Antidepressants are the primary treatment for PSD patients. However, longer treatment cycles and more side effects affect the compliance of PSD patients (2). Additionally, an increasing number of PSD patients develop tolerance to antidepressants (2). These PSD patients require a safer and simpler treatment.

Statins could affect the incidence of PSD of stroke patients (3–6). Different studies have reached distinct conclusions about the effect. One Asian study suggests that statins increase the incidence rate of PSD (4). Other studies have shown the opposite result, indicating that statins decrease the incidence rate of PSD (5, 6). The various effects of statins and different types of studies could partly explain these contradictory conclusions (7). Several studies have demonstrated that reduced testosterone levels may elevate the risk of PSD (8, 9). Therefore, low-dose statins may offer greater benefits to PSD patients, as they exert a less pronounced effect on testosterone levels (8, 9).

Statins may have varying effects on PSD depending on the stage of stroke. The use of statins before a stroke may reduce the risk of PSD, whereas post-stroke use could potentially increase it (10, 11). Thus, identifying an effective treatment strategy for patients in the acute phase of stroke with PSD is essential. Our analysis of a retrospective cohort, utilizing unsupervised machine learning methods, revealed that stroke patients receiving statins during the acute phase in conjunction with rTMS exhibited a lower incidence of PSD. Several studies have indicated that high-frequency rTMS is an effective treatment for PSD, particularly for patients in the acute phase of stroke (12–14). rTMS may counteract the adverse effects of statins in acute PSD patients, while statins could provide longer-term benefits for those in the chronic phase of PSD. Consequently, combining statins with rTMS may provide a comprehensive solution to these challenges.

This study aims to explore whether the combination of statins and rTMS can decrease PSD in ischemic stroke patients. Additionally, we aim to analyze further whether the combination of low-dose statins and rTMS better affects PSD patients.

## Methods

### Cohort 1: retrospective explored cohort

#### Patients

The study cohort was a retrospective observational cohort. The cohort comprised consecutive patients with ischemic stroke. Patients were recruited from the Neurology and Rehabilitation Departments of the Affiliated Hospital of Youjiang Medical University for Nationalities between January 1, 2018, and May 30, 2019.

The inclusion criteria included ischemic stroke patients aged 18 years or older who had undergone head CT or MRI examinations, met the WHO ischemic stroke diagnostic criteria and received conventional medicine and rehabilitation therapy post-admission. The exclusion criteria were as follows: (1) patients with a recent history of depression before onset; (2) patients unable to be evaluated for depression due to aphasia, disturbance of consciousness, cognitive disorder, or other conditions; (3) patients who taken other lipid-lowering drugs such as fenofibrate; (4) patients with intracerebral hemorrhage, subarachnoid hemorrhage, or severe systemic disease; (5) Patients who are intolerant to statins or rTMS treatment; (6) patients who withdrew from the study or could not provide outcome events.

#### Data collected

Baseline patient data were collected from electronic medical records, including demographic information, vital signs, and laboratory data. The data collected at admission included age, gender, heart rate, and blood pressure. Critical laboratory data collected at admission included PLT (platelet), INR (international normalized ratio), ALT (alanine aminotransferase), AST (aspartate aminotransferase), CRP (C-reactive protein), among others. Blood lipid levels, including TC (total cholesterol), TG (triglycerides), HDL-C (high-density lipoprotein cholesterol), and LDL-C (low-density lipoprotein cholesterol), were recorded at admission and discharge. Comorbidities, such as renal insufficiency, epilepsy, and pneumonia, were also documented. Medical histories and medication profiles were obtained using structured questionnaires completed by patients or their relatives. Two experienced neurologists, blinded to the patient’s conditions and outcomes, collected and evaluated these data.

#### Post-stroke depression

The diagnosis of post-stroke depression (PSD) was identified using ICD-9-CM codes 296.2, 296.3, 300.4, or 311. The Mini International Neuropsychiatric Interview (MINI) (6–8), a structured diagnostic psychiatric interview based on the Diagnostic and Statistical Manual of Mental Disorders, 4th Edition (DSM-IV), was also used. According to these criteria, patients were diagnosed with major depression if they presented one core symptom and at least four additional depressive symptoms. Alternatively, patients were diagnosed with minor depression if they exhibited at least one core symptom and between two and four additional symptoms. PSD was defined as encompassing both major and minor depressive disorders.

#### Classify data by unsupervised machine learning

We utilized Python 3.8 for our analysis. For cluster analysis, we first standardized all data using the StandardScaler module from the sklearn library, and then classified data using Hierarchical Clustering methods (AgglomerativeClustering module, sklearn library). The heatmap illustrated the distinct characteristics among the classified patient groups. We compared outcome events across the different identified groups. Grouping can yield clinical significance by clarifying differences in PSD among patient groups. Subsequently, we applied chi-square tests and *t*-tests to further analyze the factors within the selected groups. Significant differences were found in statin use and rTMS across the Hierarchical Clustering groups. We subsequently validated the impact of statin use and rTMS on the incidence of PSD.

### Cohort 2: prospective validated cohort

#### Study subjects

This study was a prospective cohort study. Ischemic stroke patients were recruited from the neurology and rehabilitation departments of affiliated hospitals of Youjiang Medical University for Nationalities. Patients were enrolled from January 2020 to December 2022 and followed up until December 2023.

Patients who took statins after admission were assigned to the statin group and the remaining patients were assigned to the control group. The study included only patients who did not take statins before admission. Patients were prescribed statins and specific types based on the guidelines for the management of stroke (15).

The inclusion criteria and exclusion criteria were similar as cohort 1.

#### Baseline data

Baseline patient data were similar as cohort 1.

We evaluated patients’ NIHSS (National Institutes of Health Stroke Scale) scores and mRS (Modified Rankin Scale) scores at admission. Two experienced neurologists, blinded to patients’ conditions and outcomes, collected and evaluated these clinical scale scores.

#### Statin use

We collected data on patients’ statin use during hospitalization and follow-up. Low-dose and medium-high-dose statins were defined according to the ACC/AHA guidelines on statin intensity (16). The types of statins used in our cohort included atorvastatin, rosuvastatin, simvastatin, and others. Statins were classified into lipophilic (atorvastatin, simvastatin, pitavastatin, lovastatin) and hydrophilic (rosuvastatin) categories. For patients whose statin type or dosage was adjusted during their course of treatment due to changes in their condition, we typically record the statin type or dosage used at the first administration during hospitalization. If the duration of statin use after the change in type or dosage exceeds 70% of the total usage time, the changed type or dosage will be recorded. Lower statin compliance was defined as using statins continuously for less than 40% of the follow-up period. The cohort did not include patients with lower statin compliance. When patients experience significant side effects from statin use and need to discontinue the medication, they are withdrawn from the cohort.

#### rTMS

Multiple rTMS protocols have been proposed for treating post-stroke depression; this study adopted the treatment approaches from several well-powered studies with robust conclusions (12–14). All patients underwent rTMS therapy for 4 weeks, with five sessions per week, totaling 20 sessions. rTMS was administered to the left dorsolateral prefrontal cortex (DLPFC) at 10 Hz, with magnetic stimulation strength set at 80–100% of the motor threshold level. Each daily treatment included 20 sequences (20 min), each consisting of 4 s of continuous stimulation followed by a 56-s interval (12–14). When patients experience intolerable side effects from transcranial magnetic stimulation therapy or are deemed unsuitable for further treatment by a physician, they will be withdrawn from the cohort.

#### Outcome events

The primary outcome was the incidence rate of PSD at 6- and 12-months post-onset. The secondary outcomes included favorable functional outcomes (FFO) at 6- and 12-months post-onset. A favorable functional outcome was a modified Rankin Scale (mRS) score of less than 3. Outcome events were collected post-discharge through face-to-face interviews.

### Statistical analysis

Statistical analyses were conducted using SPSS 23.0 for Windows. The threshold for statistical significance was set at *p* < 0.05.

#### Baseline character

Continuous variables (e.g., blood pressure, laboratory measures) normally distributed between two groups were analyzed using a t-test and expressed as mean ± standard deviation (SD). Continuous variables (e.g., NIHSS) that were abnormally distributed between two groups were analyzed using the Mann–Whitney *U* test and expressed as the median and interquartile range (IQR). Categorical variables (e.g., gender, history of disease) and ordinal variables (e.g., mRS) were analyzed using the chi-square test and expressed as numbers and percentages.

#### Outcome variables

A chi-square test was employed to compare the incidence rates of PSD, optimism, and FFO between the two groups. Additionally, we compared the incidence of post-stroke depression across various subgroups. These subgroups were categorized by different doses, type of statins and whether patients underwent repetitive transcranial magnetic stimulation treatment.

We employed multivariate logistic regression analysis to control for confounding factors. In cases where the extreme data exhibited significant imbalance, subgroup analysis and propensity score matching were considered for data processing. However, since the validation cohort did not show a notable imbalance in extreme risk factors, only multivariate logistic regression was used for data analysis.

Logistic regression was used to analyze the relationship between the combination of low-dose statins and rTMS, or other risk factors, and the incidence rate of PSD at different time points. Eligible factors for multivariable regression were screened as follows: (1) a *p*-value of less than 0.05 in univariable logistic regression; (2) factors selected using LASSO regression; (3) factors meeting criteria (1) and (2). The eligible data were then analyzed using multivariable logistic regression. Odds ratios (ORs), 95% confidence intervals (CIs), and *p*-values were calculated using logistic regression.

## Results

### Cohort 1

The exploratory cohort included 117 patients. Thirty-two were female (27.4%), and the mean age was 63.15 ± 11.002 years.

**Figure 1:**
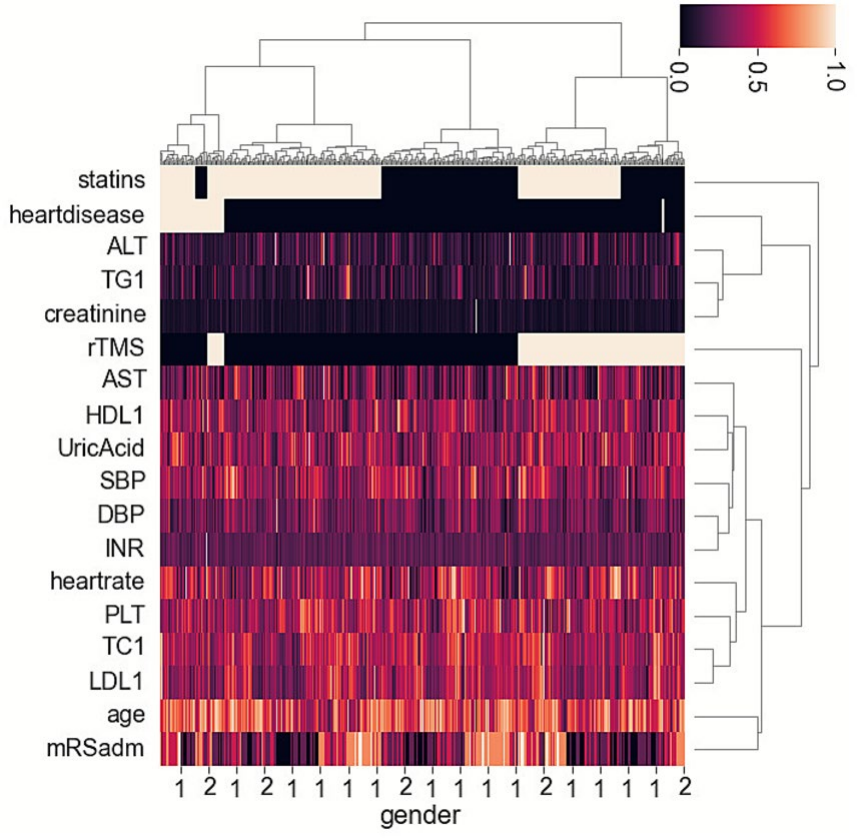
Heatmap showing hierarchical clustering methods for post-stroke depression and associated risk factors in ischemic stroke patients.

The heatmap suggested that two groups (HCgroup1 and HCgroup2) were optimal for hierarchical clustering methods (Figure 1). The heatmap also indicated significant distinctions between statins and rTMS (Figure 1). Comparing the incidence of PSD between the two groups, HCgroup1 (3.4%) had a significantly lower rate of PSD (*p* < 0.001) than HCgroup2 (17.7%).

When comparing data between HCgroup1 and HCgroup2, it was found that HCgroup1 had more patients taking statins (90.5% vs. 52.1%, *p* < 0.001), more patients using rTMS (61.9% vs. 33.3%, *p* = 0.015), fewer patients experiencing severe conditions (mRS at admission ≥ 4) (9.5% vs. 29.2%, *p* = 0.003). The results were similar to another study, indicating that patients with more severe conditions at admission had a higher incidence of PSD (15). However, the relationship between a higher rate of statin uses or rTMS and a lower rate of PSD requires further validation.

### Cohort 2

#### Baseline characteristics

Initially, we recruited 647 eligible patients for the study. Among them, 102 patients were lost to follow-up or had missing data on statin treatment and PSD. Consequently, our study included 545 patients, 433 in the statin group and 112 in the control group.

The mean age of the cohort was 60.99 ± 12.768 years, with 31.9% of patients being female (174 patients). The statin group had higher TC, TG, LDL-C, and HDL-C levels at admission than the control group. These TC, TG, and LDL-C levels in the statin group all decreased and were lower than those in the control group at discharge. Other features showed no significant differences (Table 1).

**Table 1:** Baseline characteristic and outcome data by univariate analysis.

Risk factor	Statin group (*N* = 433)	Control group (*N* = 112)	P*
Baseline characteristic			
Age, years	60.52(12.7)21	62.81(12.845)	0.090
Female, %	141(32.6)	33(29.5)	0.531
Admission mRS score	1(0–4)	1(0–4)	0.613
Admission NIHSS score	3.68(3.289)	3.50(3.249)	0.602
Optimism at admission	121(27.9)	34(30.4)	0.614
rTMS treatment	160(37.0)	32(28.6)	0.098
SBP at admission, mmHg	146.82(22.703)	144.38(23.858)	0.814
DBP at admission, mmHg	86.09(15.256)	83.50(13.968)	0.134
Heart rate at admission	82.05(14.465)	82.44(13.005)	0.783
Body temperature at admission	36.49(0.438)	36.60(0.412)	0.675
History of hypertension, %	287(66.3)	76(67.9)	0.753
History of diabetes Mellitus, %	107(24.7)	31(27.7)	0.520
History of ischemic stroke, %	68(10.2)	48(10.0)	0.318
pneumonia, %	125(28.9)	30(26.8)	0.663
epilepsy, %	17(3.9)	2(2.7)	0.595
Anti-infection drug, %	141(32.6)	199(41.5)	0.531
Laboratory data at admission			
Platelet, mmol/l	204.00(62.632)	198.79(71.625)	0.142
INR	1.02(0.134)	1.06(0.228)	0.115
Hemoglobin, mmol/l	127.26(21.168)	129.09(20.641)	0.406
Albumin, mmol/l	36.49(6.551)	36.67(7.018)	0.802
ALT, mmol/l	25.323(19.111)	23.26(15.188)	0.290
AST, mmol/l	26.84(15.567)	25.62(15.956)	0.462
Creatinine, mmol/l	82.72(34.547)	89.29(72.723)	0.266
Glucose, mmol/l	7.43(3.056)	6.81(2.844)	0.052
Triglyceride, mmol/l	1.63(1.234)	1.38(0.964)	**0.044**
Total cholesterol, mmol/l	4.19(1.097)	3.74(0.703)	**<0.001**
HDL-C, mmol/l	1.26(0.369)	1.18(0.332)	**0.042**
LDL-C, mmol/l	2.53(0.919)	1.88(0.475)	**<0.001**
Laboratory data at hospital discharge			
Triglyceride, mmol/l	1.18(0.464)	1.20(0.537)	0.715
Total cholesterol, mmol/l	3.74(0.781)	3.82(0.720)	0.351
HDL-C, mmol/l	2.04(1.386)	1.94(1.251)	0.477
LDL-C, mmol/l	1.96(0.862)	2.17(0.795)	**0.019**
Outcome data at 6 months after onset			
PSD, %	79(18.2)	26(23.2)	0.235
FFO, %	295(68.1)	63(56.3)	**0.018**
Outcome data at 12 months after onset			
PSD, %	83(19.2)	27(24.1)	0.340
FFO, %	313(72.3)	67(59.8)	**0.010**

P* was calculated using ANOVA, Chi-square test, or Mann–Whitney *U* test as appropriate. NIHSS, National Institute of Health stroke scale; mRS, Modified Rankin Scale; rTMS, repetitive transcranial magnetic stimulation; SBP, systolic blood pressure; DBP, diastolic blood pressure; INR, international normalized ratio; ALT, glutamic-pyruvic transaminase; AST, glutamic oxaloacetic transaminase; HDL-C, high-density lipoprotein cholesterol; LDL-C, low-density lipoprotein; PSD, post-stroke depression; FFO, favorable function outcome. Bold font indicates *p* < 0.05, suggesting that the difference is statistically significant.

#### Chi-square test for PSD and FFO

The statin group (18.2%) exhibited a lower incidence of PSD 6 months post-onset compared to the control group (23.2%) (*p* = 0.235). The statin group (19.2%) showed a lower incidence of PSD at 12 months post-onset than the control group (24.1%) (*p* = 0.340). The statin group (68.2%) exhibited a higher rate of FFO at 6 months post-onset compared to the control group (56.3%) (*p* = 0.018). The statin group (72.3%) showed a higher rate of FFO at 12 months post-onset than the control group (59.8%) (*p* = 0.010) (Table 1).

In a more detailed subgroup, the low-dose statins with the rTMS subgroup exhibited the lowest incidence of PSD, while the medium and high-dose statins with the rTMS subgroup and the control subgroup had similar incidences of PSD. There were significant differences in the incidence of post-stroke depression between patients treated with different types of statins and those in the control group (Table 2).

**Table 2:** PSD distribution in different subgroups (%).

Subgroup	6 months	*P*	12 months	*P*
Control group	24(30.0)	–	26(32.5)	–
Difference dose of statins				
Low-dose statins and rTMS	12(9.6)	**<0.001**	13(10.4)	**<0.001**
Medium and high statins and rTMS	10(28.6)	0.877	11(31.4)	0.910
Difference type of statins				
lipophilic statins and rTMS	26(12.9)	**<0.001**	28(13.9)	**<0.001**
Hydrophilic statins and rTMS	13(10.5)	**<0.001**	12(9.7)	**<0.001**

P* was calculated using the Chi-square test. PSD post-stroke depression, and rTMS repetitive transcranial magnetic stimulation. Bold font indicates *p* < 0.05, suggesting that the difference is statistically significant.

#### Logistic regression for PSD

Univariable logistic regression indicated that the combination of low-dose statin and rTMS was associated with PSD at 6 months (OR = 0.425 [0.224–0.807], *p* = 0.009) and PSD at 12 months (OR = 0.464 [0.249–0.865], *p* = 0.016), and FFO at 6 months (OR = 1.567 [1.012–2.426], *p* = 0.044) and FFO at 12 months (OR = 1.710 [1.163–2.554], *p* = 0.031).

Multivariable logistic regression indicated higher NIHSS scores at admission (OR = 1.127, *p* < 0.001) were associated with PSD at 6 months. The combination of low-dose statin and rTMS (OR = 0.370, *p* = 0.003) and higher heart rate (OR = 0.341, *p* = 0.028) were inversely associated with PSD at 6 months. Higher NIHSS scores at admission (OR = 1.130, p < 0.001) were associated with PSD at 12 months. The combination of low-dose statin and rTMS (OR = 0.386, *p* = 0.004), higher heart rate (OR = 0.263, *p* = 0.013), and lower urea nitrogen levels (OR = 0.910, *p* = 0.034) were inversely associated with PSD at 12 months. The relationships between risk factors and FFO at 6 and 12 months are detailed in Table 3.

**Table 3:** Multivariate logistic regression for FFO.

Risk factor	OR (95%CI)	P*
FFO at 6 months		
Low dose statin and rTMS	3.240(1.668–6.293)	**0.001**
Higher NIHSS score at admission	0.468(0.400–0.548)	**<0.001**
Younger at admission	1.023(1.000–1.046)	**0.047**
Higher hemoglobin at admission	1.022(1.008–1.037)	**0.002**
Gastrointestinal hemorrhage	0.064(0.216–0.914)	**0.004**
FFO at 12 months		
Low dose statin and rTMS	2.491(1.516–4.963)	**<0.001**
Higher NIHSS score at admission	0.614(0.550–0.686)	**<0.001**
Higher TC at admission	0.789(0.625–0.996)	**0.046**
Higher hemoglobin at admission	1.018(1.005–1.030)	**0.006**
Gastrointestinal hemorrhage	0.058(0.129–0.865)	**0.033**

P* was calculated by Multivariate Logistic regression. FFO, favorable function outcome; rTMS, repetitive transcranial magnetic stimulation; NIHSS, National Institute of Health stroke scale; TC, total cholesterol; INR, international normalized ratio. Bold font indicates *p* < 0.05, suggesting that the difference is statistically significant.

## Discussion

In the study, we used unsupervised machine learning to analyze risk and protective factors for PSD in an ischemic stroke patient cohort. The heatmap of hierarchical clustering methods indicated a significant difference in the use of statins and rTMS between the two groups. The group with a lower incidence of PSD had more patients taking statins or using rTMS. The validated cohort analysis revealed the statins group exhibited a lower incidence of PSD and a higher incidence FFO at 6 and 12 months. Comparing subgroup data, the low-dose statins with the rTMS subgroup had the lowest incidence of PSD, while the medium- and high-dose statins subgroup had the highest incidence of PSD. The combination of low-dose statin and rTMS was associated with lower PSD and higher levels of FFO at both 6 and 12 months.

In a study, we utilized unsupervised machine learning to explore factors influencing the prognosis of ischemic stroke patients undergoing transcranial magnetic stimulation therapy (17). In cohort 1, Using unsupervised machine learning to identify risk factors for PSD, we found that the use of statins among stroke patients was linked to a lower incidence of post-stroke depression. Moreover, stroke patients taking statins who also received rTMS exhibited an even lower incidence of depression. This suggests that combining both treatments may have a synergistic effect in reducing post-stroke depression. Therefore, we validated this conclusion in cohort 2, which served as the validation cohort.

The cohort 2 suggested that the statins group had a better prognosis. Our previous studies also reached a similar conclusion regarding the combination of statins and rTMS for ischemic stroke patients (17). However, we found a complex association when we analyzed the relationship between PSD and statins. This complexity was particularly evident when we analyzed the distribution of PSD in different subgroups by taking statins and using rTMS. The low-dose statins subgroup and the combination of low-dose statins and rTMS showed more significant benefits in decreasing PSD. However, the medium and high-dose statin subgroups exhibited a reversed effect, increasing PSD. The dosage of statins affected the incidence of PSD. This conclusion is not supported by similar research, as prior studies have not performed a detailed analysis of the impact of different statin dosages on post-stroke depression. Some studies concluded that different types of statins had varying effects on PSD (6, 18). Our study did not observe any differences in the effects among various types of statins, which may be due to differences in patient numbers, regional distribution across studies, and the higher proportion of patients in our study using low-dose statins.

The pleiotropic effects of statins might cause a complex relationship. Statins have an anti-inflammatory effect (7). Some studies suggest that brain inflammation following a stroke is related to symptoms of PSD (3, 19). However, the anti-inflammatory effects were not the only mechanisms by which statins influence PSD. Additionally, the lipid-lowering effects of statins also have an impact on PSD (8, 9). One study found that low cholesterol levels are related to post-stroke emotional disturbances (9). Another study suggested that low cholesterol levels might be related to the incidence of PSD (8). Therefore, the different effects of statins could have a comprehensive relationship with PSD. Low-dose statins with a lower lipid-lowering effect might be more beneficial for PSD patients.

Some studies found that rTMS reduced PSD (13, 14). Additionally, rTMS decreased PSD through various mechanisms, such as adjusting connectivity alterations in the depression circuit (2, 12). Therefore, rTMS and statins had specific mechanisms for reducing PSD. The combination of statins and rTMS exhibited an additive effect in the mechanism. rTMS is only administered to patients during hospitalization. Therefore, the effect of rTMS on PSD may not be persistent. The effect of statins could be long-lasting due to their convenience for patients after discharge. Therefore, the combination of statins and rTMS had an additive effect at different stages of stroke recovery. This is particularly evident in the combination of low-dose statins and rTMS, as demonstrated by the study’s results.

Our study had several limitations. First, this study is a cohort study, which may have selection bias. Therefore, we conducted multivariate analysis and subgroup analysis of confounding factors to ensure the reliability of the conclusions. Second, the number of patients in the medium to high-dose statins and rTMS subgroup and the rTMS-only subgroup was smaller. However, the statistical difference remained significant. Therefore, the results remain credible. Finally, the effects of rTMS in combination with different treatment parameters may vary. The duration of the effects following rTMS treatment also requires further investigation. Therefore, further research is needed to explore whether varying treatment protocols influence the combination treatment.

In conclusion, taking statins decreases the incidence of PSD in ischemic stroke patients. Patients in the statins combined with the rTMS group had a lower incidence of PSD. Combining low dose statins and rTMS could reduce PSD and further improve the prognosis of ischemic stroke patients. The conclusions of this study require further validation through large-scale, multicenter clinical trials.

## Data Availability

The raw data supporting the conclusions of this article will be made available by the authors, without undue reservation.
