# Supplementary material for: Low-dose statins combined with repetitive transcranial magnetic stimulation reduce post-stroke depression
Source: Front Neurol. 2025 Nov 26;16:1649263. doi: 10.3389/fneur.2025.1649263 (PMC12689410; doi:10.3389/fneur.2025.1649263)
Supplement: Supplementary file 1 [file Table_1.DOCX]

**Supplementary Table 01.** Multivariate Logistic regression for FFO and optimism

| Risk factor | OR (95%CI) | P* |  |
| --- | --- | --- | --- |
| **FFO at 6 months** | | |  |
| low dose statin and rTMS | | 3.240(1.668-6.293) | **0.001** |
| Higher NIHSS score at admission | | 0.468(0.400-0.548) | **<0.001** |
| Younger at admission | | 1.023(1.000-1.046) | **0.047** |
| Higher haemoglobin at admission | | 1.022(1.008-1.037) | **0.002** |
| Gastrointestinal haemorrhage | | 0.064(0.216-0.914) | **0.004** |
| **FFO at 12 months** | | |  |
| low dose statin and rTMS | 2.491(1.516-4.963) | **<0.001** |  |
| Higher NIHSS score at admission | 0.614(0.550-0.686) | **<0.001** |  |
| Higher TC at admission | 0.789(0.625-0.996) | **0.046** |  |
| Higher haemoglobin at admission | 1.018(1.005-1.030) | **0.006** |  |
| Gastrointestinal haemorrhage | 0.058(0.129-0.865) | **0.033** |  |

P* was calculated by Multivariate Logistic regression. FFO favourable function outcome, rTMS repetitive transcranial magnetic stimulation, NIHSS National Institute of Health stroke scale, TC total cholesterol, INR international normalized ratio.
